# CD31-associated vascular phenotyping using Doppler ultrasound and dual-energy CT for recurrence risk stratification in papillary thyroid cancer

**DOI:** 10.1186/s40644-025-00975-w

**Published:** 2025-12-10

**Authors:** Yan Zhou, Feng Xu, Yu Hu, Xiao Li, Yan Si, Guoyi Su, Feiyun Wu, Xiaoquan Xu

**Affiliations:** 1 Department of Radiology, The First Affiliated Hospital with Nanjing Medical University, No. 300, Guangzhou Rd, Gulou District, Nanjing, China; 2 Department of Medical Imaging, The Affiliated Suqian First People’s Hospital with Nanjing Medical University, Suqian, China; 3 Department of Ultrasound, The First Affiliated Hospital with Nanjing Medical University, Nanjing, China; 4 Department of Pathology, The First Affiliated Hospital with Nanjing Medical University, Nanjing, China; 5 Department of Thyroid Surgery, The First Affiliated Hospital with Nanjing Medical University, Nanjing, China

**Keywords:** Papillary thyroid cancer, Doppler ultrasound, Multidetector computed tomography, Angiogenesis, Recurrence

## Abstract

**Background:**

Angiogenesis plays a pivotal role in PTC aggressiveness and recurrence. CD31, an endothelial marker, provides a histological correlate of tumor vascularity. This study aimed to establish a multimodal imaging framework using Doppler ultrasound (US) and dual-energy CT (DECT) to capture CD31-associated vascular phenotypes for recurrence risk stratification.

**Methods:**

A total of 414 PTC patients (training set, 151; test set 1, 137; test set 2, 126) were included. Clinical and radiographic features, including demographics, tumor morphology, Doppler US grade, US grayscale values, and DECT parameters, were analyzed. CD31 expression was assessed by immunohistochemistry in the training set and served as a reference for vascular phenotyping. Logistic regression was applied to identify imaging-derived vascular signatures linked to CD31 expression. Recurrence-free survival (RFS) was analyzed across all datasets to evaluate prognostic value.

**Results:**

High CD31 expression correlated with advanced tumor stage (*p* = 0.003), lymph node metastasis (*p* = 0.001), extrathyroidal extension (*p* < 0.001), and recurrence (*p* < 0.001). The vascular phenotype model integrating Doppler US grade, unenhanced electron density (ED), arterial-phase iodine concentration (IC), and venous-phase IC/ED ratio achieved an AUC of 0.815 (95% CI: 0.744–0.874) for discriminating CD31 expression in the training set. This imaging-derived phenotype was significantly associated with RFS in the training set (*p* = 0.017), test set 1 (*p* = 0.006), and test set 2 (*p* = 0.002), effectively identifying patients at higher risk of recurrence.

**Conclusion:**

Preoperative vascular phenotyping with Doppler US and DECT enables noninvasive assessment of CD31-associated angiogenic activity and enhances recurrence risk stratification in PTC.

**Supplementary Information:**

The online version contains supplementary material available at 10.1186/s40644-025-00975-w.

## Introduction

Papillary thyroid carcinoma (PTC) is generally associated with a favorable prognosis. However, a subset of patients experiences recurrence, leading to clinical challenges and the need for more intensive management [1–3]. Accurate identification of patients at high risk of recurrence is clinically valuable, as it informs surgical planning, supports tailored treatment strategies, and allows for optimized follow-up schedules, ultimately improving patient outcomes [2–5].

In current clinical workflows, recurrence risk in PTC is typically assessed using postoperative pathological findings and biomarkers, offering limited value in the preoperative setting. Additionally, tumor heterogeneity poses a major challenge to reliable risk stratification. To bridge this gap, researchers have explored preoperative clinical and imaging features, such as patient demographics, Hashimoto’s thyroiditis, and ultrasound (US) characteristics including microcalcifications and irregular margins [6–12]. However, the predictive performance of these indicators remains insufficient, highlighting the need for more reliable, biologically informed, and non-invasive approaches to early risk stratification.

Angiogenesis plays a pivotal role in tumor growth, invasion, and metastasis by ensuring an adequate blood supply. CD31 (PECAM1), an endothelial marker essential for angiogenesis, facilitates neovascularization and has been implicated in tumor progression across multiple cancers [12–16]. In PTC, elevated CD31 expression is associated with increased angioinvasion and aggressive tumor behavior, correlating with a higher likelihood of recurrence [17–19]. However, CD31 expression typically requires postoperative immunohistochemistry (IHC), which restricts its use as a preoperative biomarker. This underscores the need for imaging-based approaches that can noninvasively capture tumor vascular characteristics reflective of CD31 expression.

Doppler US and dual-energy CT (DECT) provide complementary strengths for vascular phenotyping in thyroid cancer. Doppler US visualizes intratumoral blood flow, whereas DECT quantifies parameters such as iodine concentration, which correlate with microvascular density [20–30]. These modalities offer a non-invasive means of capturing angiogenesis-related imaging phenotypes, potentially serving as surrogates for histological markers like CD31. Previous studies have demonstrated associations between contrast-enhanced US or CT-derived vascular parameters and CD31-positive microvessel density in various tumor types [31–33]. Yet, no study has systematically integrated Doppler US and DECT to characterize CD31-associated vascular phenotypes and evaluate their prognostic implications in PTC.

**Fig. 1:**
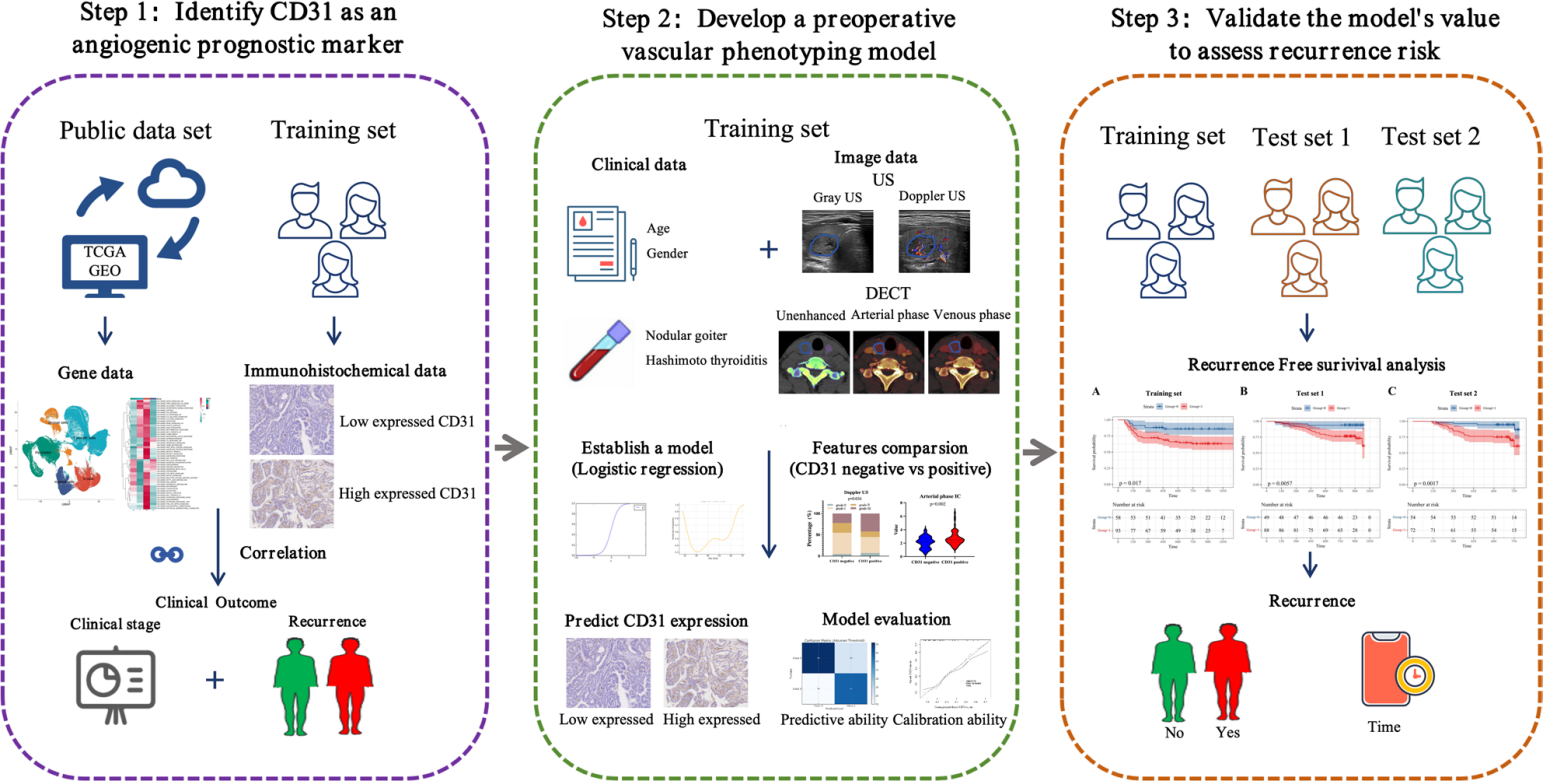
Study workflow. Overview of the study design. The workflow included three steps: (1) evaluation of CD31 as a prognostic marker and biological reference of angiogenesis in PTC; (2) development of an imaging-derived vascular phenotyping model using Doppler US and DECT features validated against CD31 expression; and (3) validation of the vascular phenotyping model for recurrence risk stratification

Therefore, this study had three objectives: (1) to examine the relationship between CD31 expression and invasive clinicopathological features as well as recurrence in PTC; (2) to establish a multimodal imaging model that characterizes vascular phenotypes associated with CD31 expression using Doppler US and DECT parameters; and (3) to evaluate the prognostic value of these imaging-derived phenotypes for recurrence-free survival (RFS) across multiple datasets. By bridging imaging-based vascular phenotyping with histological validation, this approach provides a non-invasive framework for refined risk stratification in PTC. The study workflow is illustrated in Fig. 1.

## Methods

### Public data

Single-cell transcriptomic data of PTC were obtained from the GEO database (GSE184362) [34]. After quality control, batch effects were corrected using the Harmony algorithm, followed by clustering to identify malignant cell populations. Tumor cells were further subclustered to characterize highly invasive subpopulations, and gene set variation analysis (GSVA) was performed to investigate pathways related to tumor aggressiveness, with a particular focus on angiogenesis.

STAR-counts data and clinical information for PTC were retrieved from The Cancer Genome Atlas (TCGA) portal (https://portal.gdc.cancer.gov). The data were converted to TPM format and normalized using a log2(TPM + 1) transformation. After filtering for samples with both RNA sequencing data and clinical information, 429 samples were selected, including 158 normal controls, 142 stage I-II PTC patients, and 129 stage III-IV PTC patients. We analyzed PECAM1 (CD31) expression levels across these groups and performed survival analyses to compare RFS between low and high PECAM1 (CD31) expression groups, evaluating its potential as a prognostic marker for recurrence risk in PTC.

### Patients

The study, approved by our hospital’s Ethics Committee (2022-SRFA-035), waived written informed consent due to its retrospective design. A total of 2,689 consecutive patients with clinically suspected PTC from June 2019 to June 2022 were initially enrolled. Patients were eligible for inclusion: (1) age ≥ 18 years; (2) histologically confirmed PTC; and (3) underwent both preoperative US and CT imaging within one month prior to surgery. A total of 2,440 patients met these criteria. The exclusion criteria were as follows: (1) patients who did not undergo standardized radical surgery (*n* = 282); (2) missing clinical data (*n* = 138); (3) tumor lesions smaller than 1 cm (*n* = 1082), as sub-centimeter lesions preclude accurate measurement of imaging parameters; (4) unclear visualization of lesions on DECT and US (*n* = 107); (5) incomplete pathological information (*n* = 167); (6) missing follow-up data (*n* = 190); (7) patients with evidence of persistent disease (*n* = 56). After applying these criteria, the dataset was divided according to surgical dates. The training set consisted of 155 patients who underwent surgery between June 2019 and June 2020. CD31 IHC was performed on tissue samples from this cohort, and 4 samples with poor quality staining were excluded. The test set 1 included 137 patients who underwent surgery between June 2020 and June 2021, and the test set 2 comprised 126 patients operated on between June 2021 and June 2022.

**Fig. 2:**
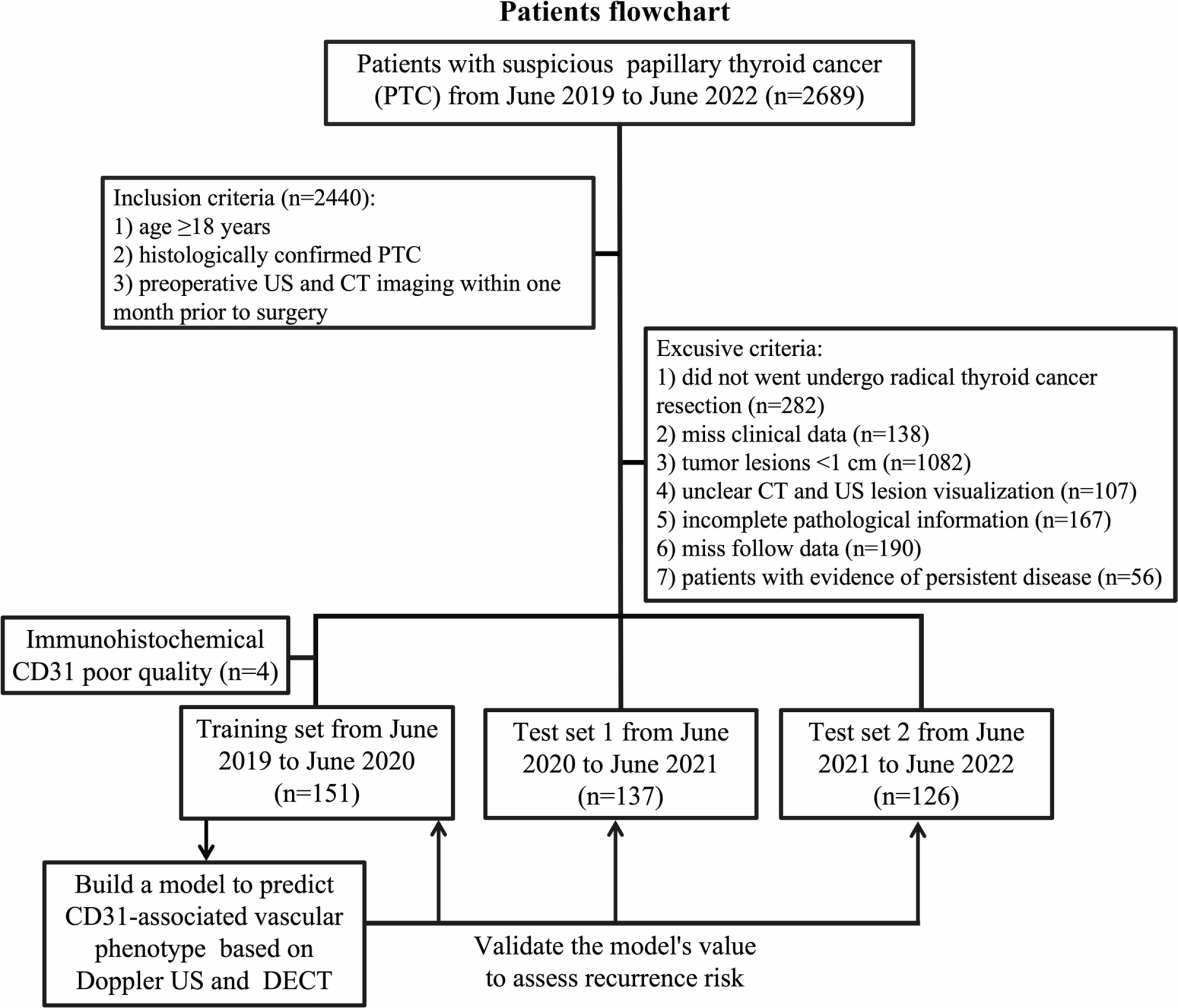
Patient flowchart. Flow diagram of patient selection and dataset division. The final cohort included 151 patients in the training set, 137 patients in test set 1, and 126 patients in test set 2

An imaging-based vascular phenotyping model was developed in the training set using angiogenesis-related Doppler US and DECT parameters, with CD31 expression serving as the biological reference. The prognostic value of this vascular phenotype was then evaluated in all three datasets by analyzing its association with RFS. A schematic overview of patient selection and dataset usage is shown in Fig. 2.

### Clinical data

Clinical data were obtained from medical records, including age, gender, history of nodular goiter, history of Hashimoto’s thyroiditis, and thyroid function. TNM stage and extrathyroidal extension (ETE) were determined according to clinical and postoperative pathological findings. Information on postoperative treatments, including radioactive iodine (RAI) ablation and thyroid-stimulating hormone (TSH) suppression therapy, was also collected for all eligible patients.

### Follow-up protocol and definition of recurrence

Patients were followed every 3–6 months in the first year and annually thereafter. Recurrence was defined based on clinical evidence, which included biochemical, structural, and functional signs of disease after the initial surgery according to 2025 ATA recommendations [2–4, 35] and included: (1) structural recurrence, defined as new loco-regional or distant lesions detected on imaging (US, CT, MRI, PET/CT, or radioiodine scintigraphy) with cytologic or histologic confirmation when feasible; (2) functional recurrence, defined as new abnormal radioiodine uptake compatible with disease on diagnostic or post-therapy scans, or metabolically active disease on PET/CT; and (3) biochemical recurrence, defined as persistent or rising serum thyroglobulin (Tg) levels (suppressed Tg ≥ 1 ng/mL or TSH-stimulated Tg ≥ 10 ng/mL) or rising anti-thyroglobulin antibody (TgAb) levels in the absence of structural or functional evidence, after excluding assay interference and residual normal thyroid tissue. All recurrence events were adjudicated through multidisciplinary team discussion, integrating comprehensive clinical, biochemical, and imaging data.

### Imaging techniques

Ultrasound imaging was performed using the Siemens Acuson S3000 Color Doppler US System equipped with an L6-18 MHz linear probe. A routine B-mode thyroid examination was first conducted to evaluate gland size, morphology, echogenicity, and any suspicious lesions. Subsequently, Doppler US was employed to assess intranodular blood flow, with standardized Color Doppler settings including a pulse repetition frequency of 0.8–1.2 kHz, gain adjusted to 60–70%, and a 50 Hz wall filter to minimize motion artifacts. Blood flow patterns were visualized and graded on a four-level scale (Grade 0: no flow; Grade I: peripheral/minimal flow; Grade II: moderate intranodular flow; Grade III: marked/diffuse vascularity) [36].

DECT scans utilized a dual-source Somatom Force scanner (Siemens Healthcare). Non-contrast, arterial (25s), and venous (50s) phase acquisitions were obtained after injecting 75 mL iopromide (3.5 mL/s). Syngo Dual Energy software generated mixed images, iodine maps, and spectral datasets [22, 25]. Technical parameters are detailed in Supplemental S1.

### Tumor qualitative features

Two thyroid US specialists assessed conventional and Doppler US features, while two radiologists analyzed DECT morphological characteristics. All evaluators were blinded to clinical outcomes. For multi-lesion cases, only the largest lesion was analyzed. Inter-observer agreement was quantified by Cohen’s kappa [37]. Tumor morphological features analyzed in the study are provided in Supplemental S2.

### Tumor quantitative parameters

Quantitative Doppler US and DECT parameters were extracted to characterize vascular phenotypes associated with angiogenesis. Regions of interest (ROIs) were manually delineated separately on US and DECT images at the largest cross-sectional plane of each lesion, covering the entire solid component while excluding calcified and cystic areas. Grayscale values from routine US images were assessed for each lesion. A separate ROI was placed over the ipsilateral common carotid artery (CCA) to normalize the iodine concentration (IC). IC was quantified on iodine maps from unenhanced, arterial, and venous phase images. Normalized iodine concentration (NIC) was calculated using the following equation: NIC = IC_lesion_/IC_CCA_. The effective atomic number (Z) was assessed using unenhanced, arterial, and venous phases Z maps. Additionally, the electron density (ED) was measured from ED maps. The IC per unit of ED was then calculated, which reflects the relative IC adjusted for the tissue’s ED. The IC/ED was calculated using the following formula: IC/ED = IC_lesion_/ED_lesion_. The dual energy index (DEI) was also computed. Spectral curves were generated, and Hounsfield Units (HUs) were measured on unenhanced, arterial, and venous phases 40 keV and 70 keV images. To evaluate the energy spectrum, the slope (λ) was determined using the equation: λ= (HU_40kev_-HU_70kev_)/(70kev-40kev) [25–27]. The analyses were conducted using the average values of these parameters obtained from the two physicians, and inter-reader agreement was evaluated with intraclass correlation coefficient (ICC) [38]. The ROI delineation process and representative DECT images for parameter extraction are illustrated in Fig**. ****S1**.

### CD31 IHC analysis

CD31 expression served as the biological reference standard for validating imaging-derived vascular phenotypes. Tissue sections were deparaffinized, rehydrated, and stained with an anti-CD31 antibody. Slides were digitized into whole-slide images (WSIs) using a high-resolution scanner, followed by preprocessing (noise reduction, color normalization, and contrast adjustment). CD31 staining intensity was quantified using the HALO digital pathology platform by measuring the optical density of positively stained regions. Staining was classified as weak (1+), moderate (2+), or strong (3+) based on predefined optical density thresholds, and quantified using the H-score formula: H-Score (CD31) = (1×% weak) + (2×% moderate) + (3×% strong), where the percentages of cells with weak, moderate, and strong staining intensities were determined by the software [19, 33]. The optimal cutoff for dichotomizing CD31 expression into negative (low) and positive (high) groups was determined by the maximally selected chi-square method using the maxstat package in R, which maximizes the separation in RFS. This cutoff, established in the training set, was subsequently applied to the test sets for validation. All results were validated by an experienced oncologic pathologist (10-year expertise).

### Establish and evaluate a vascular phenotyping model

Significant univariate imaging predictors (*p* < 0.05) were entered into a multivariable logistic regression model (stepwise selection) to construct an imaging-derived vascular phenotyping score. This composite score was referenced against CD31 immunohistochemistry as the biological standard for angiogenesis. Associations were expressed as odds ratios (ORs) with 95% confidence intervals (CIs), and multicollinearity was evaluated using the variance inflation factor (VIF, threshold = 10). To further validate predictor stability, a sensitivity analysis was performed using least absolute shrinkage and selection operator (LASSO) logistic regression with 10-fold cross-validation. Model performance was assessed using receiver operating characteristic (ROC) analysis (AUC), with the 95% CIs for the AUC derived from bootstrap internal validation (1000 repetitions). Additional metrics included accuracy from confusion matrices, precision-recall balance, and Hosmer-Lemeshow calibration.

### Model validation for recurrence risk assessment

The conventional model was developed using Cox proportional hazards regression with stepwise feature selection incorporating standard clinical (including TNM stage) and tumor qualitative features. Both conventional and CD31-based models were analyzed for recurrence assessment. Patients were stratified into low-risk and high-risk groups based on the predictive models. For each model, Kaplan-Meier curves compared RFS between different risk groups with log-rank testing.

### Statistical analysis

Statistical analyses were performed using SPSS, MedCalc, and R software. Data normality was assessed by the Kolmogorov-Smirnov test. Normally distributed data were expressed as mean ± SD (independent t-test), non-normal data as median (IQR) (Mann-Whitney U test), and categorical variables by χ2 test. Significance was set at *p* < 0.05 (two-tailed).

## Results

### Prognostic value of CD31 in PTC

Single-cell RNA sequencing of PTC identified major cell populations, including thyrocytes, B cells, T/NK cells, myeloid cells, and stromal cells (Fig. S2A). Initial clustering of tumor cells yielded ten transcriptionally distinct subgroups (Fig. S2B). Based on similarity in gene expression patterns, these were subsequently consolidated into four major clusters (labeled 0–3) for downstream analyses. Among them, subgroup 2 exhibited the most aggressive transcriptomic profile, characterized by high expression of invasion- and metastasis-related genes (Fig. S2C) and enrichment of hallmark pathways linked to tumor aggressiveness, with angiogenesis ranking as the top enriched pathway (Fig. S2D).

Analysis of the TCGA cohort showed that PECAM1 (CD31) expression was significantly higher in stage III-IV tumors compared to stage I-II tumors (*p* = 0.022), and both were higher than in normal thyroid tissue (*p* < 0.05) (Fig. S2E). Kaplan-Meier analysis revealed that patients with high PECAM1 expression had significantly worse prognosis than those with low expression (*p* = 0.003; HR = 5.05, 95% CI: 1.71–14.9) (Fig. S2F), supporting the link between CD31 and aggressive tumor behavior.

**Fig. 3:**
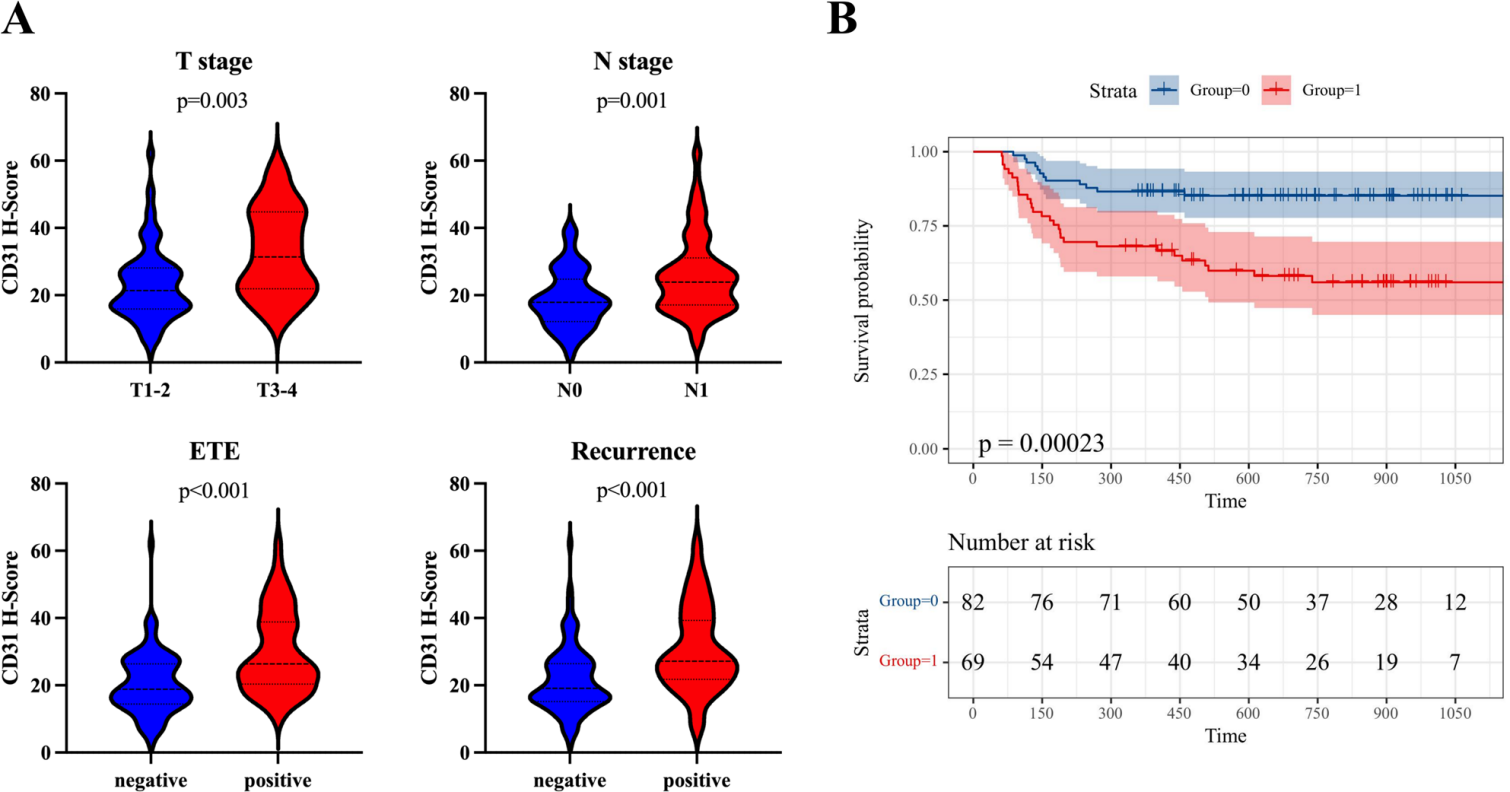
Prognostic significance of CD31 in PTC. (**A**) Violin plots showing higher CD31 H-scores in patients with advanced T stage (T3-4), nodal metastasis (N1), positive ETE, and recurrence (all *p* < 0.01). (**B**) Kaplan–Meier curves showing significantly shorter RFS in patients with high CD31 expression compared with low expression (*p* < 0.001)

The optimal H-score cut-off for CD31 expression was 21 (determined by the maximally selected chi-square method). Representative cases of CD31-negative and CD31-positive staining patterns are shown in Fig. S3. IHC analysis in our training cohort confirmed that high CD31 expression was associated with advanced T stage (*p* = 0.003), N stage (*p* = 0.001), positive ETE (*p* < 0.001), and recurrence (*p* < 0.001) (Table S1; Fig. 3A). Patients with high CD31 expression had significantly shorter RFS than those with low expression (*p* < 0.001) (Fig. 3B). Collectively, these multimodal findings establish CD31 as a robust prognostic marker of recurrence risk and provide a biological reference for imaging-based vascular phenotyping in PTC.

### Clinical and radiographic features associated with vascular phenotypes

**Fig. 4:**
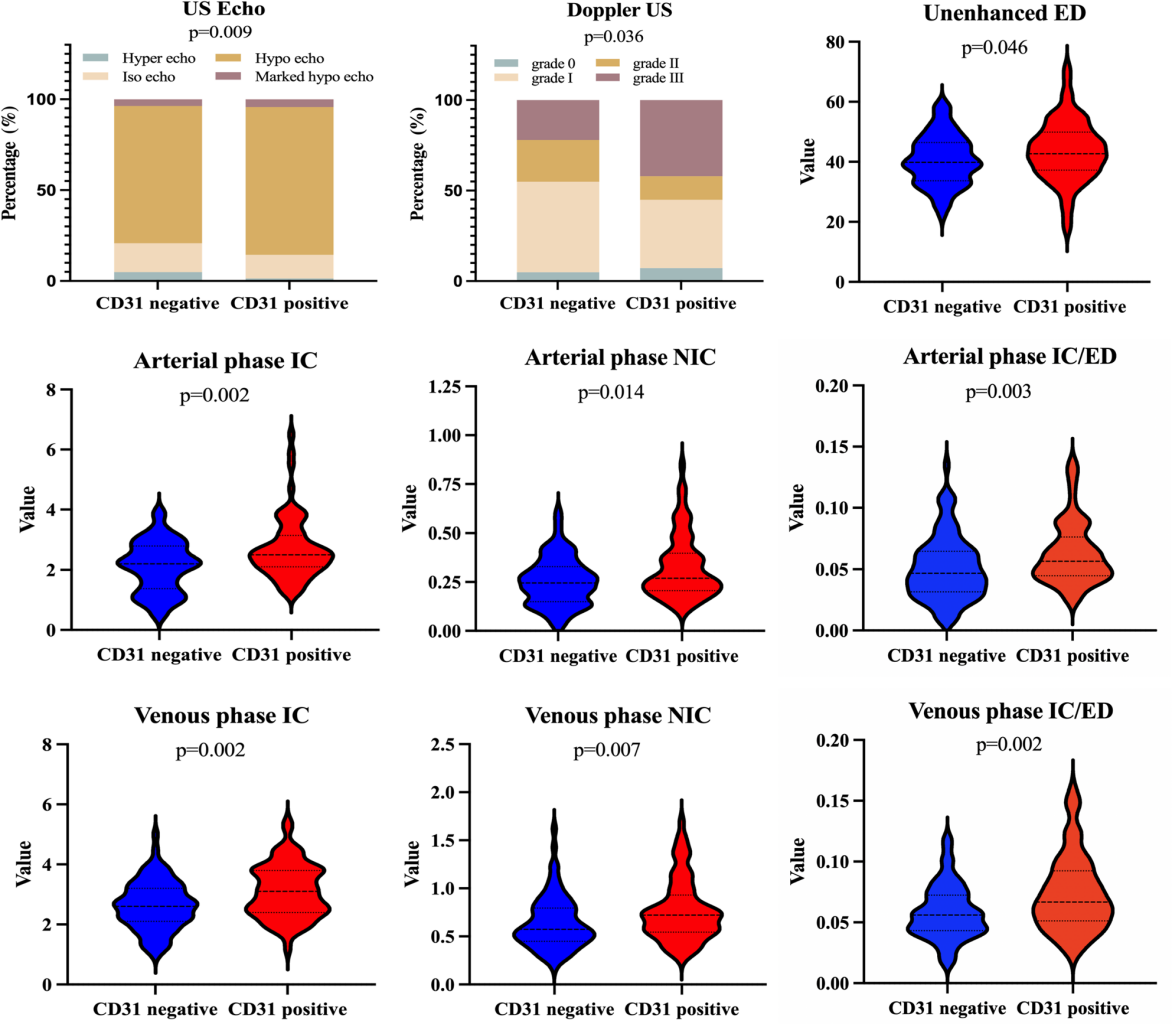
Imaging features associated with CD31-validated vascular phenotypes. CD31-high tumors demonstrated significantly more hypoechoic echogenicity (*p* = 0.009), higher Doppler US vascular grade (*p* = 0.036), and elevated DECT-derived parameters, including unenhanced ED, arterial and venous phase IC, NIC, and IC/ED ratios (all *p* < 0.05). These parameters collectively characterize imaging-derived vascular phenotypes associated with CD31 expression

The clinicopathological characteristics of patients in the training and two test sets are summarized in Table S2. All patients underwent total thyroidectomy, with 28% receiving postoperative RAI therapy and 85% receiving TSH suppression therapy. Excellent agreement was observed between two radiologists for all morphological tumor features (all kappa > 0.80, Table S3). In US evaluations, CD31-positive tumors were significantly more likely to present with hypoechoic or markedly hypoechoic echogenicity (*p* = 0.009). Moreover, a higher proportion of CD31-positive tumors were classified as grade III on Doppler US vascular grading (*p* = 0.036). No significant associations were observed for other conventional morphological features (Tables 1 and 2). Figure 4 illustrates the distribution of echogenicity and Doppler grades according to different vascular phenotype status. These findings highlight distinct vascular phenotypes associated with high CD31 expression.

**Table 1:** Comparison of clinical characteristics in CD31 negative and positive patients

Clinical characteristics	CD31 negative (82)	CD31 positive (69)	*p* value
Age (years, mean ± SD)	38 ± 11	35 ± 11	0.154
Gender			0.940
Male	29 (35.4%)	24 (34.8%)	
Female	53 (64.6%)	45 (65.2%)	
Nodular goiter			0.222
Negative	43 (52.4%)	43 (62.3%)	
Positive	39 (47.6%)	26 (37.7%)	
Hashimoto thyroiditis			0.095
Negative	56 (68.3%)	38 (55.1%)	
Positive	26 (31.7%)	31 (44.9%)	

Data are numbers of patients and parentheses indicate the proportion if not specified

**Table 2:** Comparison of radiographic characteristics in CD31 negative and positive patients

Radiographic Characteristics	CD31 negative (82)	CD31 positive (69)	*p* value
Size (mm, mean ± SD)	17.4 ± 6.9	17.9 ± 8.4	0.661
Location			0.943
Right lobe	45 (54.9%)	36 (52.2%)	
Left lobe	30 (36.6%)	27 (39.1%)	
Isthmus	7 (8.5%)	6 (8.7%)	
Site			0.292
Superior	27 (32.9%)	22 (31.9%)	
Medium	22 (26.9%)	26 (37.7%)	
Inferior	33 (40.2%)	21 (30.4%)	
Position			0.755
Ventral	22 (26.8%)	15 (21.7%)	
Medium	41 (50.0%)	36 (52.2%)	
Dorsal	19 (23.2%)	18 (26.1%)	
Aspect ratio			0.551
≤1 (wider than tall)	57 (69.5%)	51 (73.9%)	
> 1 (taller than wide)	25 (30.5%)	18 (26.1%)	
Shape			0.772
Regular	17 (20.7%)	13 (18.8%)	
Irregular	65 (79.3%)	56 (81.2%)	
Internal composition			0.371
Solid	64 (78.1%)	58 (84.1%)	
< 50% cystic	17 (20.7%)	9 (13.0%)	
> 50% cystic	1 (1.2%)	2 (2.9%)	
Echogenicity			0.009
Hyperechogenicity	4 (4.9%)	1 (1.4%)	
Isoeechogenicity	13 (15.9%)	9 (13.0%)	
Hypoechogenicity	62 (75.5%)	56 (81.3%)	
Marked hypoechogenicity	3 (3.7%)	3 (4.3%)	
Capsule contact > 25%			0.670
Negative	55 (67.1%)	44 (63.8%)	
Positive	27 (32.9%)	25 (36.2%)	
Calcification			0.061
No calcification	49 (59.8%)	36 (52.2%)	
Macrocalcification	23 (28.0%)	30 (43.5%)	
Microcalcification	10 (12.2%)	3 (4.3%)	
Doppler Ultrasound grade			0.036
Grade 0	4 (4.9%)	5 (7.2%)	
Grade I	41 (50.0%)	26 (37.7%)	
Grade II	19 (23.1%)	9 (13.0%)	
Grade III	18 (22.0%)	29 (42.1%)	

Data are numbers of patients and parentheses indicate the proportion if not specified

### Quantitative imaging parameters of CD31-associated vascular phenotypes

The agreement between two radiologists for assessing US gray scale and all quantitative DECT-derived parameters was good (all ICC > 0.75, Table S3). Comparison of imaging parameters between CD31-negative and CD31-positive vascular phenotypes revealed seven significant differences. In the unenhanced phase, ED values were significantly higher in the CD31-positive group (*p* = 0.046). During the arterial phase, CD31-positive tumors showed elevated IC (*p* = 0.002), NIC (*p* = 0.014), and IC/ED ratio (*p* = 0.003). Similarly, in the venous phase, IC (*p* = 0.002), NIC (*p* = 0.007), and IC/ED ratio (*p* = 0.002) were significantly higher in the CD31-positive group. No significant differences were detected for other parameters. These results (Table 3; Fig. 4**)** support the use of these parameters as non-invasive imaging surrogates of vascular phenotyping validated by CD31 expression.

**Table 3:** Comparison of quantitative parameters in CD31 negative and positive patients

Quantitative parameters	CD31 negative (82)	CD31 positive (69)	*p* value
Ultrasound gray scale	65.350 (45.200-70.430)	67.890 (51.310–89.160)	0.219
Unenhanced IC	0.400 (0.100–0.600)	0.400 (0.100–0.600)	0.959
Unenhanced NIC	1.000 (1.000-1.525)	1.000 (1.000–2.000)	0.308
Unenhanced Z	7.705 (7.618–7.843)	7.760 (7.625–7.865)	0.329
Unenhanced ED	39.800 (33.675–46.400)	42.700 (37.250–49.900)	0.046
Unenhanced IC/ED	0.009 (0.003–0.017)	0.009 (0.002–0.015)	0.614
Unenhanced DEI	0.005 (0.002–0.008)	0.006 (0.003–0.009)	0.246
Unenhanced λ	0.709 (0.346–1.078)	0.835 (0.472–1.193)	0.253
Arterial phase IC	2.200 (1.375-2.800)	2.500 (2.100–3.150)	0.002
Arterial phase NIC	0.245 (0.149–0.329)	0.269 (0.205–0.397)	0.014
Arterial phase Z	8.680 (8.225–9.085)	8.650 (8.275–8.985)	0.684
Arterial phase ED	44.400 (37.575–51.300)	42.700 (38.850–50.550)	0.987
Arterial phase IC/ED	0.047 (0.032–0.065)	0.057 (0.045–0.076)	0.003
Arterial phase DEI	0.028 (0.016–0.036)	0.026 (0.017–0.036)	0.731
Arterial phase λ	4.267 (2.515–5.466)	3.820 (2.516-5.700)	0.665
Venous phase IC	2.600 (2.100–3.200)	3.100 (2.400–3.800)	0.002
Venous phase NIC	0.575 (0.450–0.795)	0.722 (0.544–0.961)	0.007
Venous phase Z	8.910 (8.660–9.225)	8.880 (8.615–9.225)	0.799
Venous phase ED	47.200 (42.550–52.400)	45.000 (39.900–49.700)	0.076
Venous phase IC/ED	0.056 (0.043–0.072)	0.067 (0.051–0.092)	0.002
Venous phase DEI	0.032 (0.024–0.042)	0.031 (0.024–0.042)	0.828
Venous phase λ	4.900 (3.799–6.296)	5.108 (3.849–6.483)	0.450

Data are median and parentheses indicate interquartile range. *IC*, iodine concentration; *NIC*, normalized iodine concentration; *Z*, effective atomic number; *ED*, electronic density; *DEI*, dual-energy index

### Development of a vascular phenotyping model

A logistic regression model was constructed to derive an imaging-based vascular phenotyping score using CD31 expression as the biological reference (Table 4). Four imaging features emerged as significant predictors of the vascular phenotype: Doppler US grade (OR = 1.204, *p* = 0.041), unenhanced ED (OR = 1.075, *p* = 0.002), arterial phase IC (OR = 1.617, *p* = 0.034), and venous phase IC/ED ratio (OR = 27.050, *p* = 0.001). The events-per-variable (EPV) ratio for the multivariable model was 17.25 (69 events with 4 predictors), exceeding the recommended threshold of 10, thus supporting model stability. Variance inflation factors (VIF) were all below 10, indicating no multicollinearity. Notably, the LASSO sensitivity analysis retained the same four features, confirming predictor stability under penalized regression (Fig. S4).

**Table 4:** Results of multivariate logistic regression analyses

Variables	β	Odds ratio (95% CI)	*p* value	VIF
Doppler Ultrasound grade	0.186	1.204 (1.004–1.464)	0.041	1.200
Unenhanced ED	0.072	1.075 (1.027–1.124)	0.002	1.186
Arterial phase IC	0.480	1.617 (1.038–2.517)	0.034	1.241
Venous phase IC/ED	3.298	27.050 (3.482-210.118)	0.001	1.279

*IC*, iodine concentration; *ED*, electronic density; *CI*, confidence intervals; *VIF*, variance inflation factor

The vascular phenotyping model demonstrated strong performance, achieving an AUC of 0.815 (95% CI: 0.744–0.874) in the training set (Table S4; Fig. S5A). The 95% CI for the AUC was derived from bootstrap internal validation with 1000 repetitions, indicating robust and stable performance. Precision–recall analysis confirmed high precision across different recall levels (Fig. S5B), and confusion matrix analysis showed reliable classification of vascular phenotypes (Fig. S5C). The calibration plot indicated good agreement between predicted and observed phenotypes (Fig. S5.D). These results confirm the robustness and accuracy of imaging-derived vascular phenotyping.

### Prognostic value of the vascular phenotyping model

The median follow-up times were 1006, 860, and 702 days for the training, test set 1, and test set 2, respectively. Recurrence rates were consistent across cohorts: 27.2% (41/151), 26.3% (36/137), and 25.4% (32/126).

The clinical model, integrating tumor size and N stage, demonstrated significant prognostic value in the training set (*p* = 0.020) but showed limited generalizability in the independent test sets, with results not reaching statistical significance (test set 1: *p* = 0.063; test set 2: *p* = 0.065) (Table S5; Fig.S6).

**Fig. 5:**
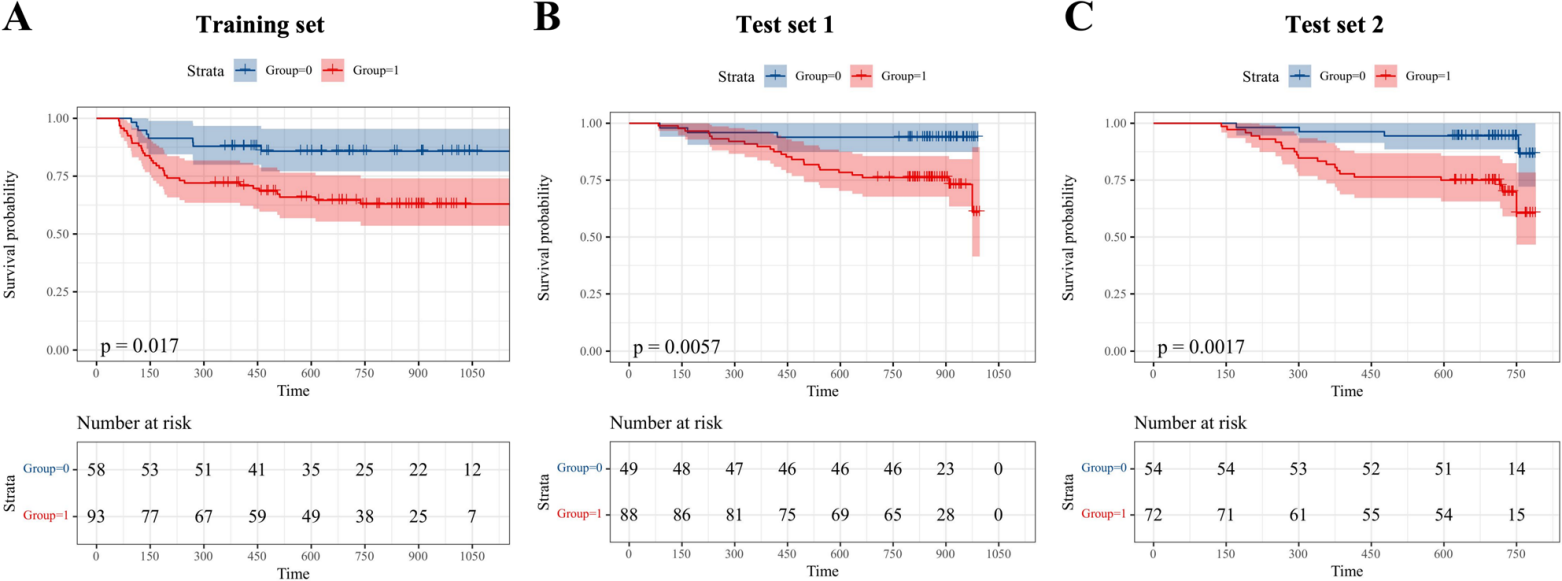
Prognostic value of the model. Kaplan–Meier analysis of RFS stratified by vascular phenotyping model predictions in (**A**) the training set (*p* = 0.017), (**B**) test set 1 (*p* = 0.006), and (**C**) test set 2 (*p* = 0.002). Patients classified into high vascular phenotype consistently exhibited worse RFS across three different datasets

**Fig. 6:**
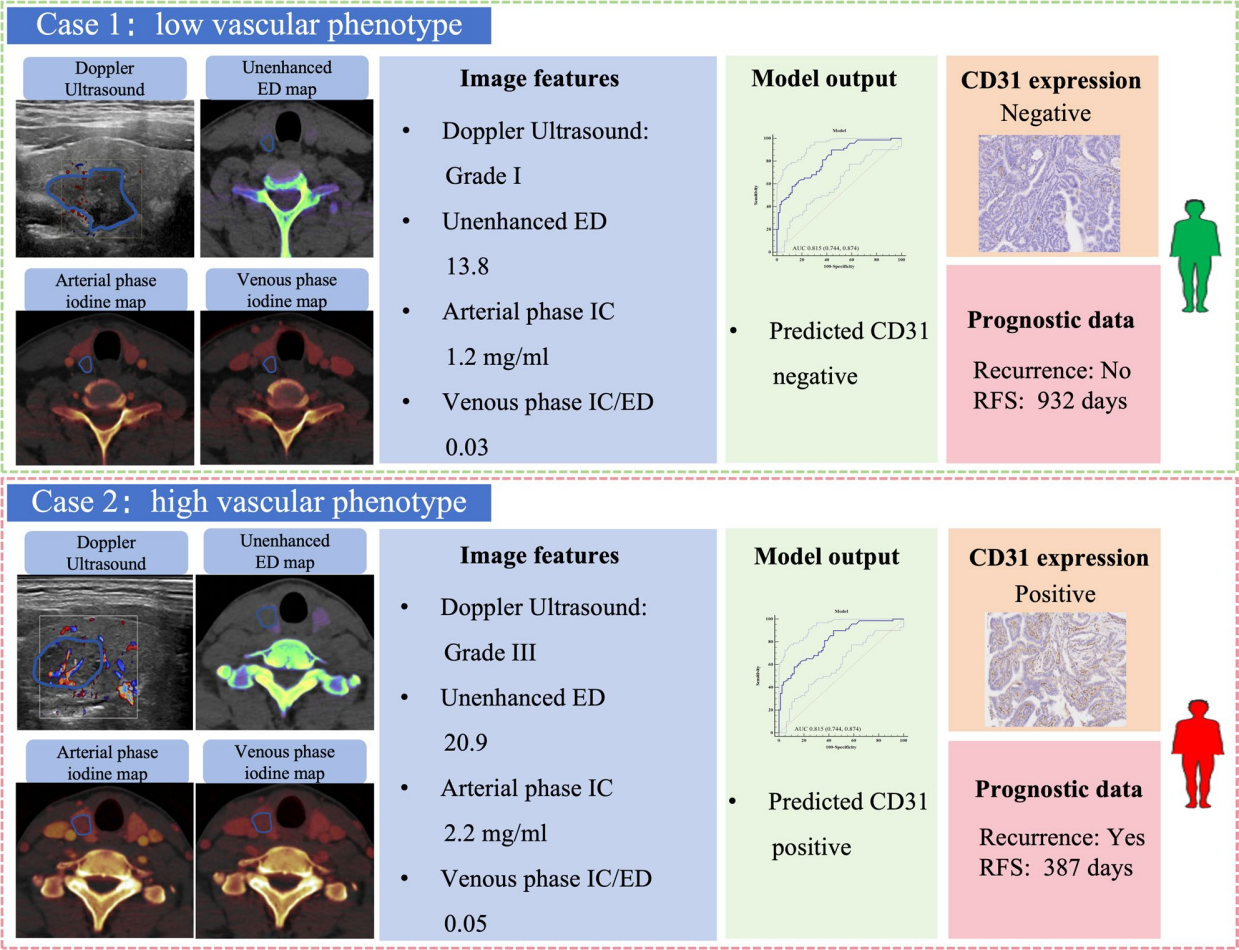
Representative clinical cases of CD31-associated vascular phenotyping. Case 1: classified as low vascular phenotype based on Doppler US and DECT features, corresponding to no recurrence with an RFS of 932 days. Case 2: classified as high vascular phenotype, associated with recurrence and an RFS of 387 days. These cases illustrate how imaging-derived vascular phenotyping can support individualized recurrence risk assessment

In contrast, the vascular phenotyping model demonstrated consistent prognostic value across all cohorts, significantly stratifying patients into high- and low-risk groups: training set (HR = 2.321, 95% CI: 1.226–4.391, *p* = 0.017), test set 1 (HR = 4.659, 95% CI: 2.109–10.293, *p* = 0.006), and test set 2 (HR = 4.666, 95% CI: 2.158–10.092, *p* = 0.002). Patients with high vascular phenotypes had significantly worse RFS (Fig. 5). Representative clinical cases further illustrated how imaging-derived vascular phenotyping could support individualized recurrence risk assessment (Fig. 6).

## Discussion

Accurate recurrence risk stratification in papillary thyroid carcinoma (PTC) remains a clinical challenge, as current prognostic tools rely heavily on postoperative pathology and demonstrate limited preoperative value [1–5]. In this study, we established a non-invasive imaging-based vascular phenotyping model, integrating Doppler US and DECT features, and validated it against CD31 expression as a biological reference of angiogenesis. By bridging vascular phenotypes with recurrence risk, our approach provides a preoperative framework to support individualized management of high-risk PTC patients.

Angiogenesis is a key driver of PTC progression and metastasis. Consistent with prior evidence linking aberrant angiogenesis to tumor aggressiveness [12–16], our single-cell RNA sequencing identified the most aggressive tumor cell subgroup as highly enriched in angiogenesis pathways, with CD31 (PECAM1) emerging as a central endothelial marker associated with vascularization, angioinvasion, and poor prognosis [17–19]. It is important to distinguish the specific role of CD31 from other key players in angiogenesis. Unlike VEGF, which primarily functions as a soluble mitogenic signal for new vessel growth, CD31 is a structural component of endothelial cell junctions that mediates cell-cell adhesion, vascular stabilization, and leukocyte trafficking. This distinct role suggests that CD31-associated vascularity may reflect a more mature, structured, and potentially invasive vascular network, as opposed to the nascent, chaotic neovasculature often driven by VEGF. Furthermore, while CD34 is a common pan-endothelial marker, its expression can also be found on hematopoietic progenitor cells. CD31, in contrast, exhibits a more specific and consistent expression on mature endothelial cells and platelets. The specific enrichment of CD31 in our most aggressive tumor subgroup underscores its potential association with a functionally adverse vascular phenotype in PTC. Our institutional analysis further confirmed CD31’s prognostic value, showing significant associations with advanced T stage, lymph node metastasis, ETE, and reduced RFS. Together, these findings establish CD31 as a reliable reference standard for vascular phenotyping in PTC.

While immunohistochemistry remains the gold standard for assessing angiogenesis, it requires postoperative tissue and thus lacks preoperative applicability. Doppler US and DECT offer complementary strengths for non-invasive vascular phenotyping. Our findings demonstrate that tumors with high CD31 expression were more likely to exhibit grade III Doppler vascularity, reflecting increased macrovascular flow, as well as elevated DECT-derived IC and IC/ED ratios, which indicate enhanced microvascular density and perfusion [22, 25–27]. These results highlight that imaging-derived vascular features can act as surrogates for CD31-associated angiogenesis, enabling their use in preoperative recurrence risk stratification.

Building on these associations, we developed a vascular phenotyping model integrating Doppler US grade, unenhanced ED, arterial phase IC, and venous phase IC/ED ratio. Each of these features captures distinct aspects of vascular biology: Doppler US grade reflects intratumoral blood flow; arterial phase IC quantifies perfusion; venous phase IC/ED ratio adjusts vascular density for tissue composition; and unenhanced ED provides contextual information on the tumor microenvironment. The model achieved strong discriminative performance, with robust calibration and balanced sensitivity and specificity. Importantly, this vascular phenotyping model consistently stratified patients by recurrence risk across all datasets, outperforming conventional clinical models based on tumor size and N stage. Patients with high vascular phenotypes exhibited significantly worse RFS, emphasizing the model’s clinical utility. Rather than directly predicting recurrence from empirical features, our approach leverages biologically grounded vascular phenotypes validated by CD31, providing mechanistic interpretability and improving clinical relevance.

This study suggests that vascular phenotyping using Doppler US and DECT can complement existing preoperative risk assessment in PTC. By providing non-invasive insights into angiogenic activity, the model may help identify patients requiring more aggressive surgical strategies, closer surveillance, or adjuvant therapies. Moreover, vascular phenotyping could be extended beyond CD31 to incorporate additional angiogenesis markers, paving the way for a broader imaging biomarker framework in thyroid and other solid tumors.

Several limitations warrant consideration. First, the retrospective single-center design with a limited sample size may restrict generalizability. Second, the exclusion of sub-centimeter lesions, while necessary for measurement accuracy, may introduce selection bias and limits the model’s applicability to larger, often more clinically consequential PTCs. Third, although the imaging parameters captured key vascular features, they may not fully represent tumor spatial heterogeneity. Fourth, region-to-region correlation between imaging features and CD31 staining was not feasible, which may introduce bias. Finally, although validated in independent test cohorts, multicenter studies with larger populations and longer follow-up are necessary to confirm robustness and clinical applicability.

In conclusion, this study demonstrates that imaging-derived vascular phenotypes, validated against CD31 expression, serve as non-invasive surrogates of angiogenesis in PTC. A Doppler US and DECT-based vascular phenotyping model shows potential to complement conventional clinical indicators for preoperative recurrence risk stratification. These findings underscore the potential of vascular phenotyping as an imaging biomarker framework to guide precision management in thyroid cancer, which merits further validation.

## Supplementary Information

Below is the link to the electronic supplementary material.

Supplementary Material 1

## Data Availability

The data are not available for public access on account of patient privacy concerns but are available from the corresponding authors if there is a reasonable request and approval from the institutional review boards of the affiliated institutions.

## Abbreviation

PTC: Papillary thyroid cancer
IHC: Immunohistochemistry
US: Ultrasound
DECT: Dual-energy computed tomography
TCGA: The Cancer Genome Atlas
RFS: Recurrence free survival
ETE: Extrathyroidal extension
TG: Thyroglobulin
TGAb: Thyroglobulin antibody
ROIs: Regions of interest
CCA: Common carotid artery
IC: Iodine concentration
NIC: Normalized iodine concentration
Z: Effective atomic number
ED: Electronic density
DEI: Dual-Energy index
HUs: Hounsfield Units
ORs: Odds ratios
CIs: Confidence intervals
VIF: Variance inflation factor
ROC: Receiver operating characteristic
AUC: Area under the curve
IQR: Interquartile range
