# Supplementary material for: CD31-associated vascular phenotyping using Doppler ultrasound and dual-energy CT for recurrence risk stratification in papillary thyroid cancer
Source: Cancer Imaging. 2025 Dec 10;26:6. doi: 10.1186/s40644-025-00975-w (PMC12801959; doi:10.1186/s40644-025-00975-w)
Supplement: Supplementary file 1 — Supplementary Material 1 [file 40644_2025_975_MOESM1_ESM.docx]

**Supplementary Material**

1. **Supplemental S1. DECT image protocol**

All DECT scans were performed using a third-generation DECT scanner (Somatom Force; Siemens Healthcare) with dual X-ray tube voltages. The protocol included 128 × 0.6 mm collimation, 0.5-second rotation time, 0.7 pitch, 256 × 256 matrix size, and a 252 × 252 mm field of view. After an unenhanced CT scan, 75 mL of iopromide (Bayer HealthCare) was injected intravenously at 3.5 mL/sec. Arterial and venous phase scans were initiated 25 and 50 seconds post-injection, respectively.

Image reconstruction was performed using a commercially available workstation with the Syngo Dual Energy software. For each neck DECT scan, three 1.5-mm slice thickness image sets were generated: 80 kVp, Sn150 kVp, and a mixed image, approximating the conventional 120 kVp CT. Iodine maps, which provide information on the content and distribution of iodine contrast within tissues, were generated at a slice thickness of 1.5 mm. Z maps and ED maps were obtained by analyzing the attenuation characteristics of tissues under dual-energy data, while energy spectrum curves were generated by plotting the CT value variations across different energy levels. Finally, the imaging workflow generated a variety of datasets, including unenhanced, arterial, and venous phase mixed images, iodine maps, Z maps, ED maps, and energy spectrum curves, which were used for subsequent analysis [1, 2].

References

1. Geng D, Zhou Y, Shang T, et al. Effect of Hashimoto's thyroiditis on the dual-energy CT quantitative parameters and performance in diagnosing metastatic cervical lymph nodes in patients with papillary thyroid cancer. Cancer Imaging. 2024;24:10.
2. Ren H, Huang J, Huang Y, et al. Nomogram based on dual-energy computed tomography to predict the response to induction chemotherapy in patients with nasopharyngeal carcinoma: a two-center study. Cancer Imaging. 2025;25:8.
3. **Supplemental S2. Tumor morphological CT features evaluation**

The following tumor morphological characteristics were evaluated: (1) size (Axial): defined as the longest diameter in the axial slice showing the maximum tumor size; (2) location: categorized as right lobe, left lobe, or isthmus; (3) site: classified as superior, middle, or inferior based on anatomical location; (4) position: categorized as ventral, middle, or dorsal in relation to the thyroid gland; (5) aspect ratio: calculated as the ratio of the longest diameter to the shortest diameter in the axial slice showing the maximum tumor size; (6) shape: defined as regular (round or nearly round) or irregular for non-regular shapes; (7) internal composition: categorized as solid, predominantly solid (<50% cystic), or predominantly cystic (>50% cystic); (8) echogenicity: classified as marked hypoechogenicity, hypoechogenicity, isoechogenicity, or hyperechogenicity in comparison to the surrounding thyroid tissue; (9) capsule contact: defined as positive if the tumor showed greater than 25% contact with the ipsilateral thyroid capsule; (10) calcification: categorized as no calcification, macrocalcification, or microcalcification. Microcalcifications were defined as calcifications with a diameter ≤ 2 mm [1, 2].

References

1. Suh PS, Baek JH, Lee JH, et al. Effectiveness of microvascular flow imaging for radiofrequency ablation in recurrent thyroid cancer: comparison with power Doppler imaging. Eur Radiol. 2024;35(2):597-607

2. Xu XQ, Zhou Y, Su GY, et al. Iodine Maps from Dual-Energy CT to Predict Extrathyroidal Extension and Recurrence in Papillary Thyroid Cancer Based on a Radiomics Approach. AJNR Am J Neuroradiol. 2022;43(5):748-755

**Table S1.** CD31 expression and its effects on clinicopathological features

| Characteristics | CD31 H-score | *p* value |
| --- | --- | --- |
| T stage |  | 0.003 |
| T1-T2 | 21.4 (15.9-28.1) |  |
| T3-T4 | 31.4 (21.9-44.8) |  |
| N stage |  | 0.001 |
| N0 | 17.9 (12.1-24.7) |  |
| N1 | 23.9 (17.1-31.1) |  |
| Extrathyroidal extension |  | <0.001 |
| Negative | 18.8 (14.4-26.4) |  |
| Positive | 26.3 (20.4-38.9) |  |
| Recurrence |  | <0.001 |
| Negative | 19.1 (15.2-26.5) |  |
| Positive | 27.2 (21.8-39.3) |  |

Data are median and parentheses indicate interquartile range.

**Table S2.** Clinicopathological characteristics of patients in the training and two test sets

| Characteristics | Training set (151) | Test set 1 (137) | Test set 2 (126) | *p* value |
| --- | --- | --- | --- | --- |
| Age (years, mean ± SD) | 37 ± 11 | 39 ±13 | 38 ± 13 | 0.128 |
| Gender |  |  |  | 0.193 |
| Male | 53 (35.1%) | 39 (28.5%) | 49 (38.9%) |  |
| Female | 98 (64.9%) | 98 (71.5%) | 77 (61.1%) |  |
| Nodular goiter |  |  |  | 0.956 |
| Negative | 86 (57.0%) | 79 (57.7%) | 74 (58.7%) |  |
| Positive | 65 (43.0%) | 58 (42.3%) | 52 (41.3%) |  |
| Hashimoto thyroiditis |  |  |  | 0.607 |
| Negative | 94 (62.3%) | 81 (59.1%) | 71 (56.3%) |  |
| Positive | 57 (37.7%) | 56 (40.9%) | 55 (43.7%) |  |
| T stage |  |  |  | 0.878 |
| T1 | 51 (33.8%) | 42 (30.6%) | 40 (31.7%) |  |
| T2 | 62 (41.1%) | 56 (40.9%) | 52 (41.3%) |  |
| T3 | 25 (16.6%) | 20 (14.6%) | 18 (14.3%) |  |
| T4 | 13 (8.5%) | 19 (13.9%) | 16 (12.7%) |  |
| N stage |  |  |  | 0.962 |
| N0 | 42 (27.8%) | 38 (27.7%) | 35 (27.8%) |  |
| N1a | 42 (27.8%) | 36 (26.3%) | 32 (25.4%) |  |
| N1b | 67 (44.4%) | 63 (46.0%) | 59 (46.8%) |  |
| M stage |  |  |  | 0.418 |
| M0 | 150 (99.3%) | 137 (100.0%) | 126 (100.0%) |  |
| M1 | 1 (0.7%) | 0 (0.0%) | 0 (0.0%) |  |
| Extrathyroidal extension |  |  |  | 0.268 |
| Negative | 93 (61.6%) | 75 (54.7%) | 66 (52.4%) |  |
| Positive | 58 (38.4%) | 62 (45.3%) | 60 (47.6%) |  |
| Recurrence |  |  |  | 0.947 |
| Negative | 110 (72.8%) | 101 (73.7%) | 94 (74.6%) |  |
| Positive | 41 (27.2%) | 36 (26.3%) | 32 (25.4%) |  |

Data are numbers of patients and parentheses indicate the proportion if not specified.

**Table S3.** Interobserver agreement for US and DECT features

| Feature | Kappa/ICC (95% CI) |
| --- | --- |
| Size | 0.782 (0.732-0.832) |
| Location | 0.852 (0.812-0.892) |
| Site | 0.843 (0.803-0.883) |
| Position | 0.861 (0.821-0.901) |
| Aspect ratio | 0.835 (0.795-0.875) |
| Shape | 0.873 (0.833-0.913) |
| Internal composition | 0.847 (0.807-0.887) |
| Echogenicity | 0.826 (0.786-0.866) |
| Capsule contact >25% | 0.854 (0.814-0.894) |
| Calcification | 0.862 (0.822-0.902) |
| Doppler Ultrasound grade | 0.841 (0.801-0.881) |
| Ultrasound gray scale | 0.791 (0.741–0.841) |
| Unenhanced IC | 0.768 (0.718–0.818) |
| Unenhanced NIC | 0.776 (0.726–0.826) |
| Unenhanced Z | 0.759 (0.709–0.809) |
| Unenhanced ED | 0.793 (0.743–0.843) |
| Unenhanced IC/ED | 0.771 (0.721–0.821) |
| Unenhanced DEI | 0.784 (0.734–0.834) |
| Unenhanced λ | 0.763 (0.713–0.813) |
| Arterial phase IC | 0.796 (0.746–0.846) |
| Arterial phase NIC | 0.788 (0.738–0.838) |
| Arterial phase Z | 0.774 (0.724–0.824) |
| Arterial phase ED | 0.802 (0.752–0.852) |
| Arterial phase IC/ED | 0.781 (0.731–0.831) |
| Arterial phase DEI | 0.779 (0.729–0.829) |
| Arterial phase λ | 0.766 (0.716–0.816) |
| Venous phase IC | 0.798 (0.748–0.848) |
| Venous phase NIC | 0.785 (0.735–0.835) |
| Venous phase Z | 0.772 (0.722–0.822) |
| Venous phase ED | 0.804 (0.754–0.854) |
| Venous phase IC/ED | 0.783 (0.733–0.833) |
| Venous phase DEI | 0.777 (0.727–0.827) |
| Venous phase λ | 0.769 (0.719–0.819) |

*IC*, iodine concentration; *NIC*, normalized iodine concentration; *Z*, effective atomic number; *ED*, electronic density; *DEI*, dual-energy index; *CI*, confidence interval.

**Table S4.** Performance of the established model used to predict CD31 status

| Statistic | Model (95% CI) |
| --- | --- |
| AUC | 0.815 (0.744-0.874) |
| Sensitivity | 0.799 (0.702-0.858) |
| Specificity | 0.661 (0.547-0.770) |
| PPV | 0.733 (0.629-0.828) |
| NPV | 0.768 (0.647-0.845) |

*AUC*, area under the curve; *PPV*, positive predictive value; *NPV*, negative predictive value.

**Table S5.** Results of Cox regression analyses for the conventional model

| Variables | β | Hazard ratio (95% CI) | Wald | *p* value |
| --- | --- | --- | --- | --- |
| Size | 0.032 | 1.032 (0.997-1.069) | 3.204 | 0.073 |
| N stage | 1.778 | 5.919 (2.940-11.913) | 24.820 | <0.001 |

*CI*, confidence interval.


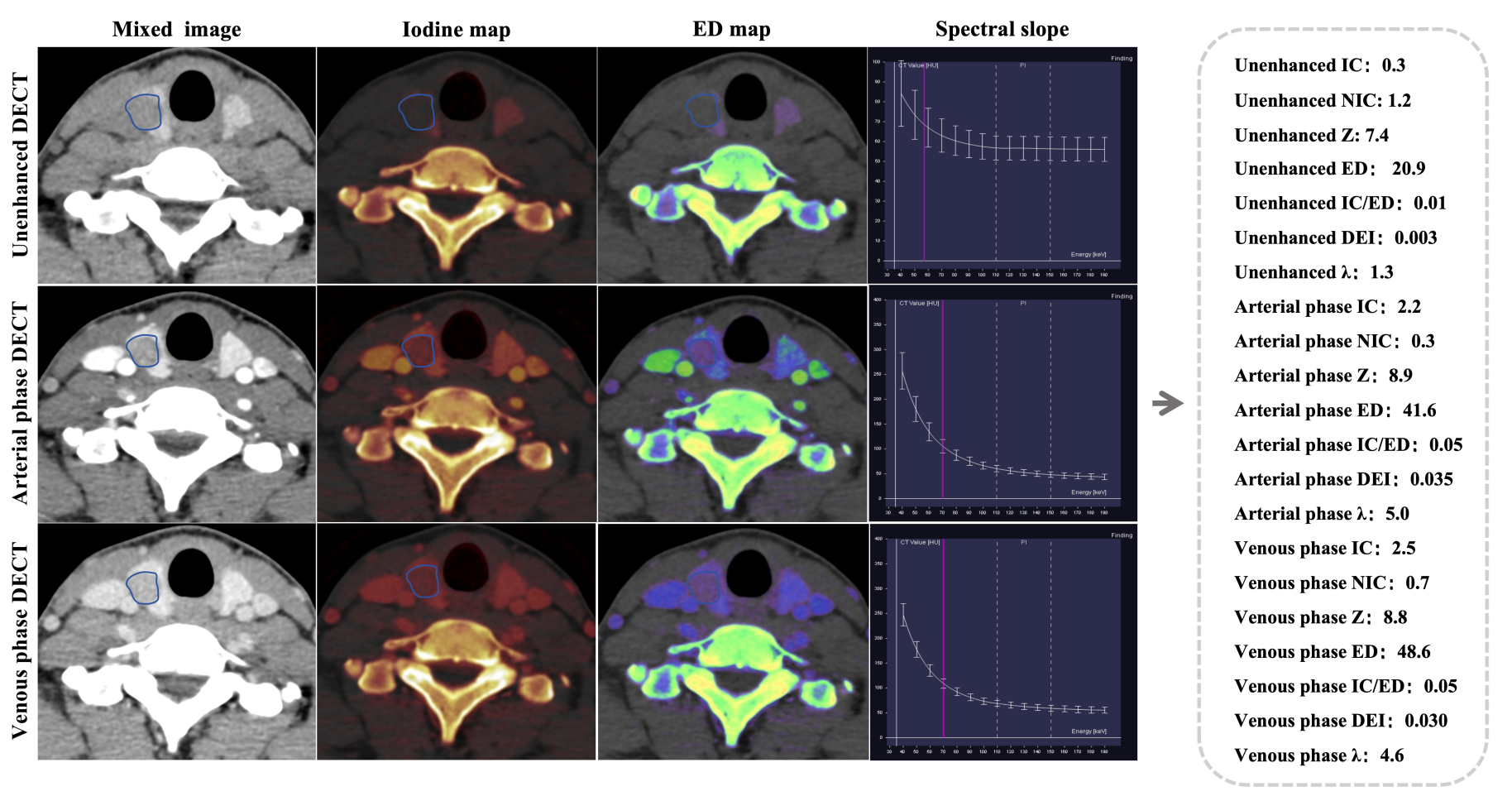


**Figure S1.** Illustration of ROI delineation and parameter extraction on dual-energy CT images. ROIs were manually drawn on the largest cross-sectional plane of the tumor to cover the solid component while avoiding calcified and cystic areas. Representative images from unenhanced, arterial, and venous phases are shown. Iodine concentration (IC) was quantified from iodine maps, normalized by the ipsilateral common carotid artery (CCA) to obtain normalized IC (NIC). Effective atomic number (Z), electron density (ED), IC/ED, dual-energy index (DEI), and spectral curve slope (λ) were also derived from corresponding maps and energy spectrum images.

**
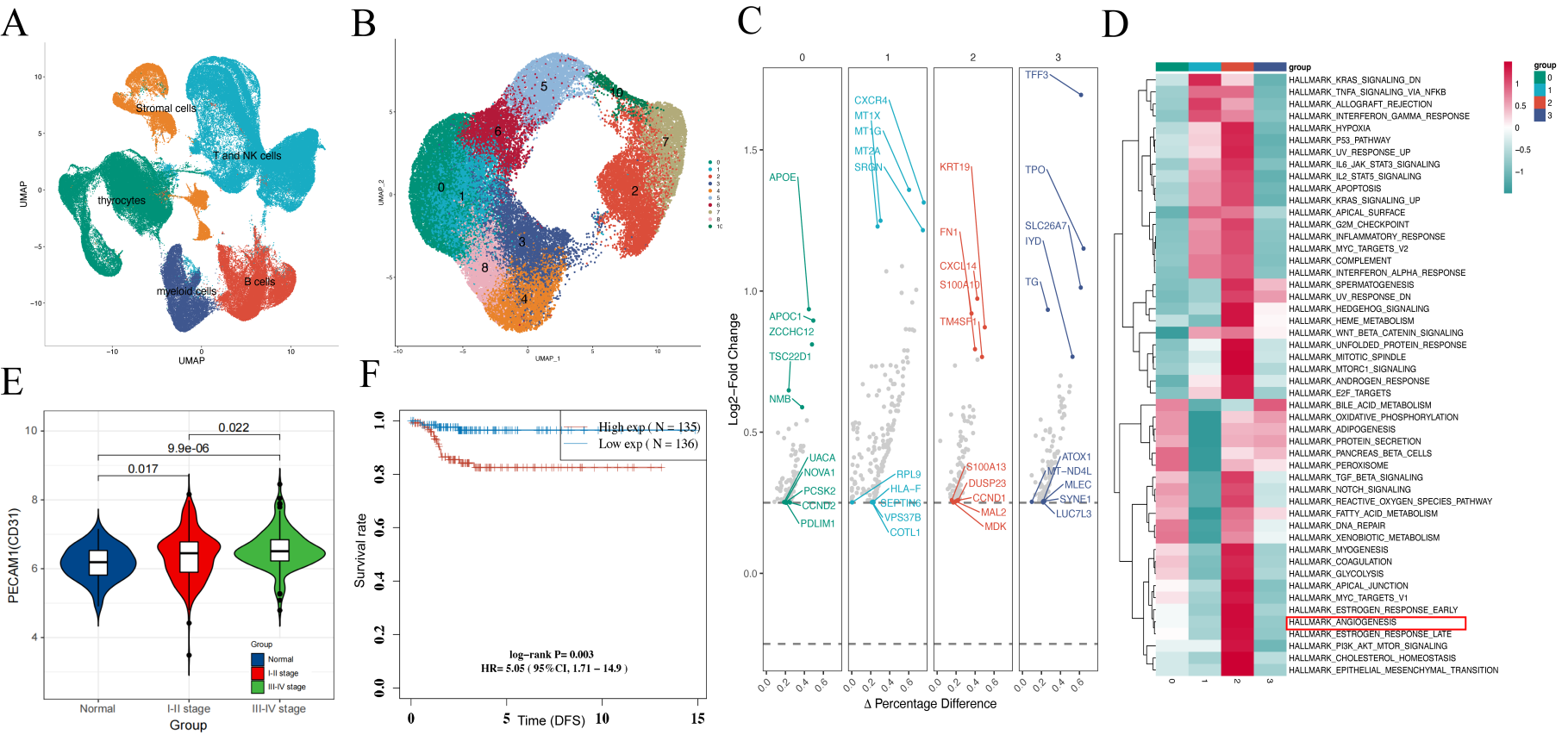
**

**Figure S2.** Single-cell transcriptomic and bulk RNA-seq analysis of PECAM1 (CD31) in PTC. (A) UMAP plot of major cell populations in PTC, including thyrocytes, B cells, T/NK cells, myeloid cells, and stromal cells. (B) UMAP plot of tumor cells after subclustering, revealing four transcriptionally distinct subgroups (0-3). (C) Heatmap of invasion- and metastasis-related gene expression across tumor subgroups, showing subgroup 2 with the most aggressive profile. (D) Hallmark pathway enrichment analysis of tumor subgroups, highlighting angiogenesis as the top enriched pathway in subgroup 2. (E) PECAM1 (CD31) expression in normal thyroid tissue and in stage I-II and stage III-IV PTC tumors from the TCGA cohort. (F) Kaplan-Meier curve revealed that patients with high PECAM1 expression had a significantly poorer prognosis than those with low expression in the TCGA cohort.


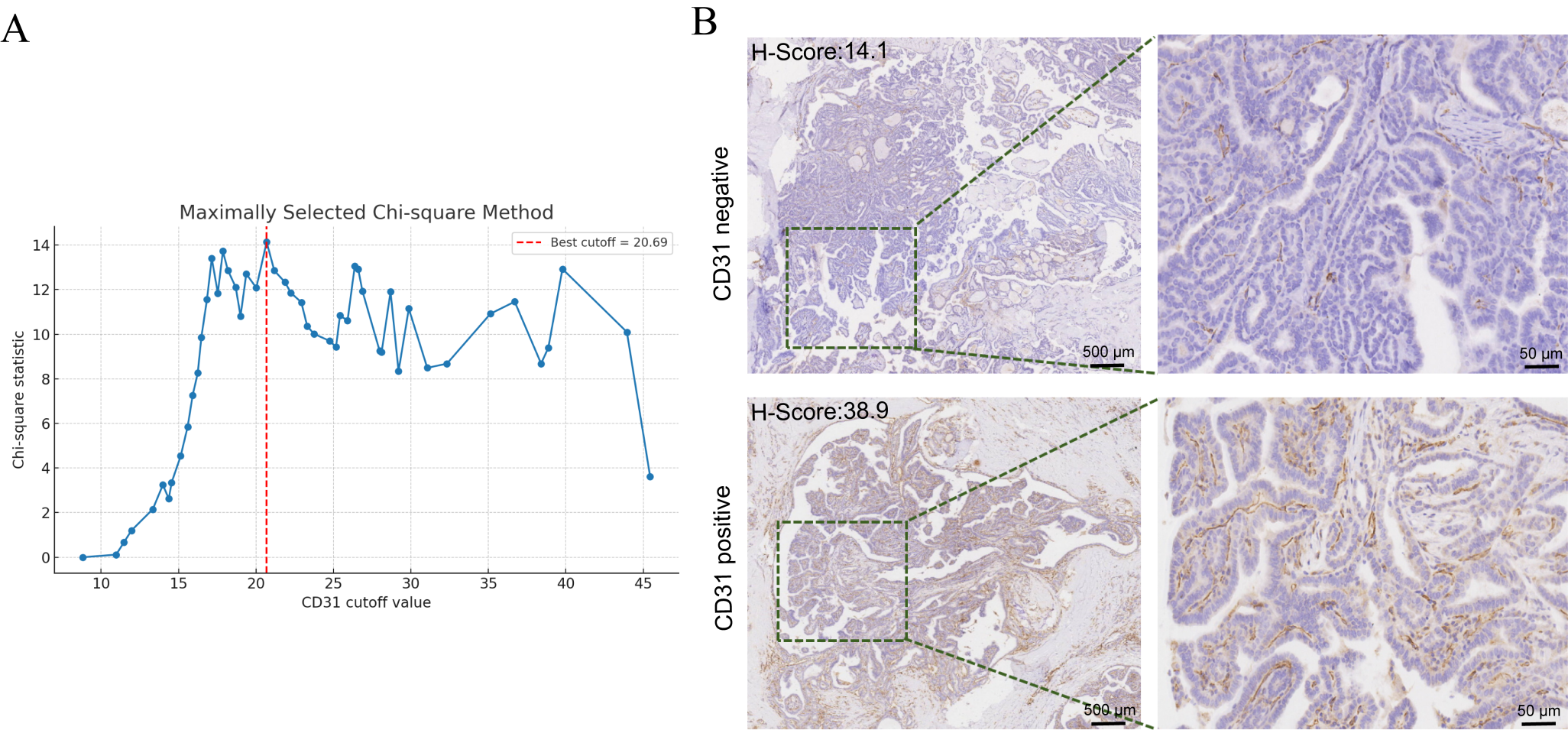


**Figure S3.** Immunohistochemical (IHC) analysis of CD31 expression in the training cohort. (A) Determination of the optimal H-score cut-off (21) for dichotomizing CD31 expression using the maximally selected chi-square method. (B) CD31 expression was localized to vascular endothelial cell membranes and cytoplasm. The CD31-negative case (H-score = 14.1) showed only a few faintly stained vessels, whereas the CD31-positive case (H-score = 38.9) displayed abundant microvessels with strong brown staining, reflecting increased vascular density.

**
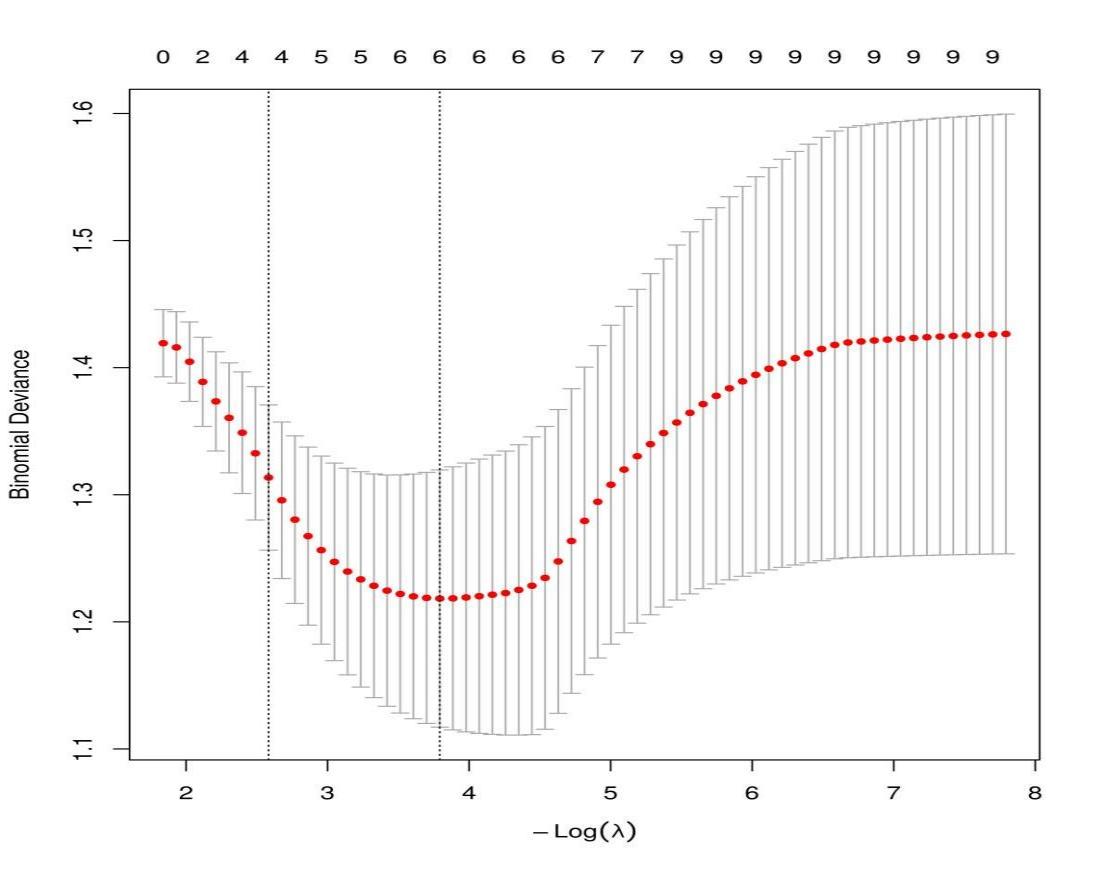
**

**Figure S4.** Feature selection and tuning parameter (λ) determination using LASSO regression with 10-fold cross-validation. The plot shows the cross-validated binomial deviance (y-axis) across a range of the log (lambda) values (x-axis). The left vertical dashed line indicates the lambda value at which the model achieves the minimum cross-validated error. The right vertical dashed line corresponds to the optimal lambda value selected by the 1-standard-error rule (λ = 0.076), which was used to build the final parsimonious model. At this optimal lambda, the model retained four non-zero coefficients, corresponding to the predictors: Doppler US grade, unenhanced ED, arterial phase IC, and venous phase IC/ED ratio.

**
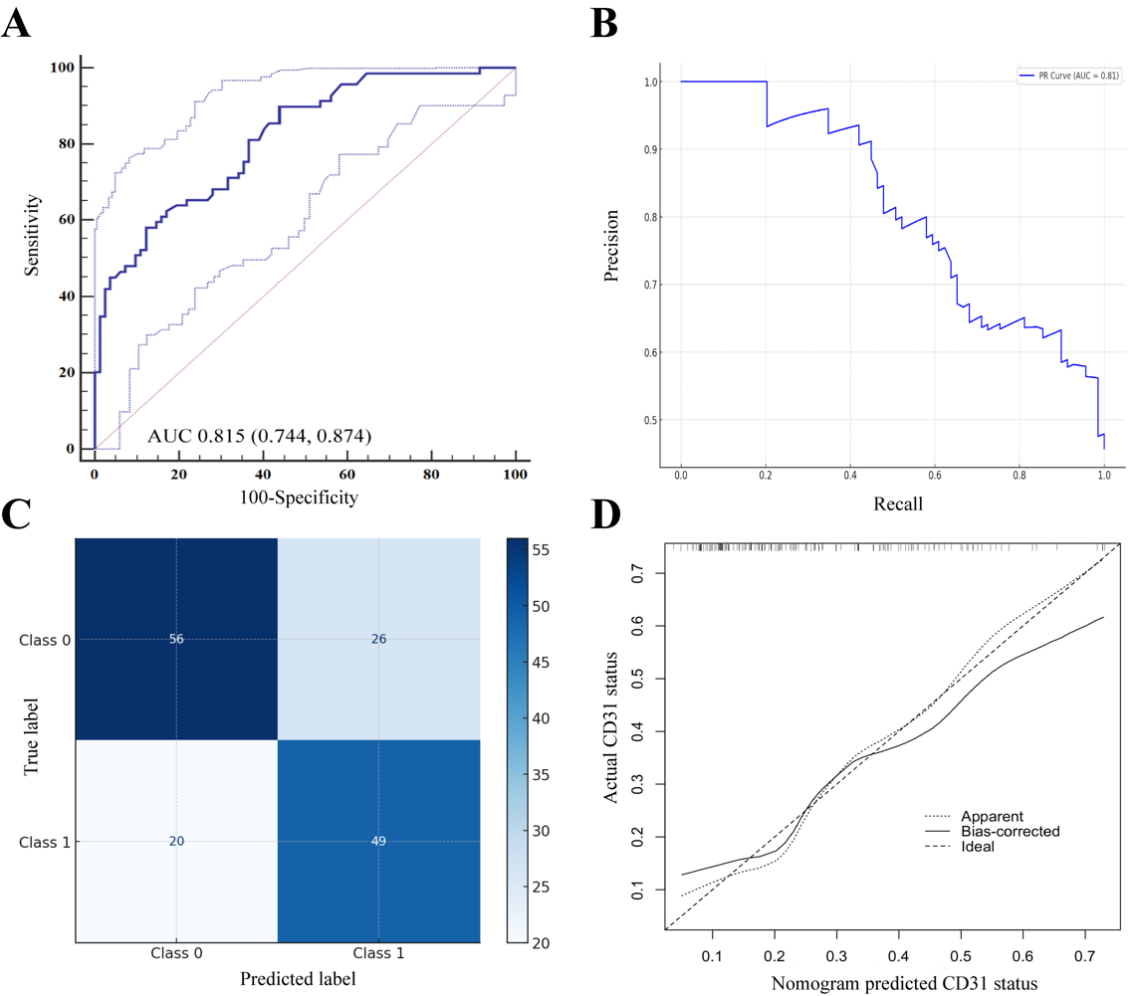
**

**Figure S5.** Performance and calibration of the vascular phenotyping model. (A) Receiver operating characteristic (ROC) curve with an area under curve (AUC) of 0.815, demonstrating strong predictive performance. (B) Precision-recall (PR) curve highlighting model precision across recall levels. (C) Confusion matrix showing model classification results at an adjusted threshold, with 56 true negatives, 49 true positives, 26 false positives, and 20 false negatives. (D) Calibration plot showing good agreement between predicted and actual CD31 status, with the bias-corrected line closely aligning with the ideal line.


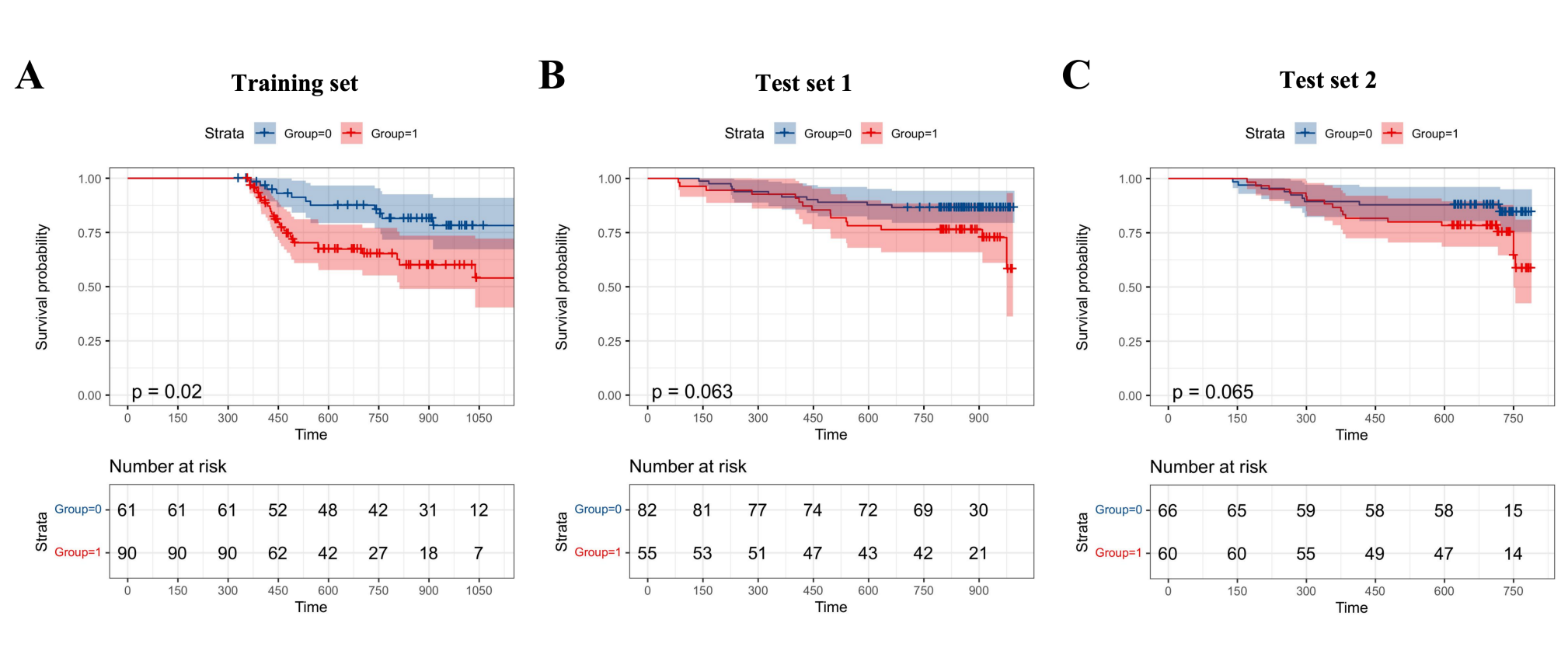


**Figure S6.** Kaplan-Meier curves illustrating recurrence-free survival (RFS) stratified by the conventional clinical model (based on tumor size and N stage) across three cohorts. (A) Training set (p = 0.020), (B) Test set 1 (p = 0.063), and (C) Test set 2 (p = 0.065). The prognostic discrimination of the model was not consistently replicated in the external validation cohorts.
